# A network model for the specification of vulval precursor cells and cell fusion control in *Caenorhabditis elegans*

**DOI:** 10.3389/fgene.2013.00112

**Published:** 2013-06-14

**Authors:** Nathan Weinstein, Luis Mendoza

**Affiliations:** Departamento de Biología Molecular y Biotecnología, Instituto de Investigaciones Biomédicas, Universidad Nacional Autónoma de MéxicoMexico City, México

**Keywords:** Caenorhabditis VPCs, vulval precursor cells, regulatory networks, discrete state network model, Caenorhabditis model

## Abstract

The vulva of *Caenorhabditis elegans* has been long used as an experimental model of cell differentiation and organogenesis. While it is known that the signaling cascades of Wnt, Ras/MAPK, and NOTCH interact to form a molecular network, there is no consensus regarding its precise topology and dynamical properties. We inferred the molecular network, and developed a multivalued synchronous discrete dynamic model to study its behavior. The model reproduces the patterns of activation reported for the following types of cell: vulval precursor, first fate, second fate, second fate with reversed polarity, third fate, and fusion fate. We simulated the fusion of cells, the determination of the first, second, and third fates, as well as the transition from the second to the first fate. We also used the model to simulate all possible single loss- and gain-of-function mutants, as well as some relevant double and triple mutants. Importantly, we associated most of these simulated mutants to multivulva, vulvaless, egg-laying defective, or defective polarity phenotypes. The model shows that it is necessary for RAL-1 to activate NOTCH signaling, since the repression of LIN-45 by RAL-1 would not suffice for a proper second fate determination in an environment lacking DSL ligands. We also found that the model requires the complex formed by LAG-1, LIN-12, and SEL-8 to inhibit the transcription of *eff-1* in second fate cells. Our model is the largest reconstruction to date of the molecular network controlling the specification of vulval precursor cells and cell fusion control in *C. elegans*. According to our model, the process of fate determination in the vulval precursor cells is reversible, at least until either the cells fuse with the ventral hypoderm or divide, and therefore the cell fates must be maintained by the presence of extracellular signals.

## 1. Introduction

**Figure 1:**
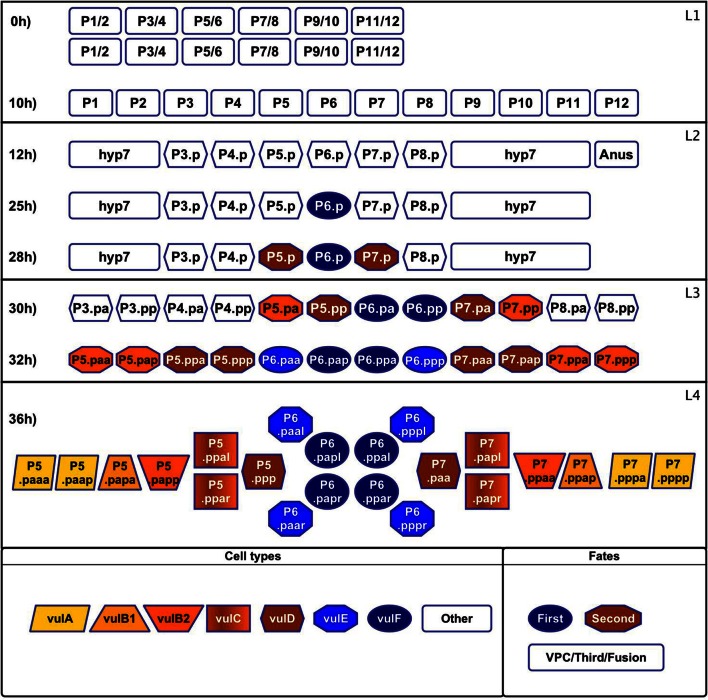
**Formation and specialization of the vulval cells during the first 36 h of development of *C. elegans***. Larval phase L1: (0 h) The worm is born with two rows of cells in the middle ventral region. (10 h) The rows merge. Larval phase L2: (12 h) The cells P1–P12 undergo a longitudinal division, the anterior daughter cells (Pn.a) become neuroblasts (not shown), while the posterior cells (Pn.p) become epidermoblasts. P3.p–P8.p become vulval precursor cells (VPCs), P1.p, P2.p, P9.p, P10.p, and P11.p fuse with hyp7 and P12.pa forms the anus. (25 h) P6.p is induced by the anchor cell to acquire the first fate and starts secreting the lateral signal. (28 h) P5.p and P7.p respond to the lateral signal of P6.p and acquire the second fate. The rest of the VPCs acquire the third fate forming the pattern 3^*rd*^3^*rd*^2^*nd*^1^st^2^*nd*^3^*rd*^. Larval phase L3: (30 h) Cells P3.p–P8.p divide longitudinally. (32 h) The descendents of the third fate fuse with hyp7 and the rest divide longitudinally again. Larval phase L4: (36 h) Formation of the adult vulval cells: some descendants of the VPCs divide a third time with the pattern LLTN TTTT NTLL. L stand for a lateral division, the resulting anterior and posterior cells append “a” and “p” to their names, respectively. T is a transverse division, the resulting left and right cells append “l” and “r” to their names, respectively. N stands for no division.

*Caenorhabditis elegans* is a nematode used extensively as a model organism for study in the areas of genomics, cell biology, neuroscience, aging, genetics, developmental biology, and cell differentiation (Hodgkin, 2005; Herman, 2006; Golden and Melov, 2007; Hobert, 2010). In particular, the vulva of *C. elegans* has been amply used in studies of organ formation, cellular fusion, and intracellular signaling (Sharma-Kishore et al., 1999; Sternberg, 2005; Félix, 2012). The vulva is a small organ with the main functions of copulation and egg laying. Anatomically, it is formed by a stack of seven different epithelial rings, namely (in ventral-to-dorsal order): vulA, vulB1, vulB2, vulC, vulD, vulE, and vulF, containing a total of 22 nuclei (Figure 1). Each of these rings is either a single tetranucleate syncytium, a binucleate syncytium (vulD) or two half-ring binucleate syncytia (vulB1 and vulB2). Despite its small size, this organ interacts with muscles, nerves, the gonad, and the ventral hypodermis (Lints and Hall, 2009).

Vulval development may be divided into three main stages; namely, formation of the VPCs (Figure 1, 0–12 h), cell specialization, and morphogenesis. During cell specialization (Figure 1, 25–36 h) the fate of the cells is determined by induction from the anchor cell (AC)—a gonadal cell located dorsally with respect to the cell P6.p–, by lateral signaling among the VPCs, and the concentration of Wnt ligands secreted by the AC and other cells near the tail of the worm. Then, during morphogenesis the vulval cells migrate towards the AC, and they fuse forming the seven rings that give the adult vulva its final shape. Furthermore, during this stage the AC breaks the membrane that separates the gonad from the epidermis, connecting both tissues and opening the vulval channel (Sharma-Kishore et al., 1999; Sternberg, 2005; Lints and Hall, 2009).

There are several models describing the process of cell specialization in the vulva of *C. elegans* (Félix and Barkoulas, 2012). The first developed models were diagrammatic (Sternberg and Horvitz, 1986, 1989), where a concentration gradient of the inductive signal determines the cell fate. Then, dynamical models were created to highlight the importance of the order in the sequence of signals (Fisher et al., 2005, 2007), while other models emphasized the importance of the inductive signal gradient (Giurumescu et al., 2006, 2009; Hoyos et al., 2011). Furthermore, some models incorporated an evolutionary perspective (Giurumescu et al., 2009; Hoyos et al., 2011), while other models were developed to test new modeling techniques (Kam et al., 2003, 2008; Sun and Hong, 2007; Li et al., 2009; Fertig et al., 2011). Importantly, none of these models explain how cell fusion is controlled during the process of fate determination, the importance of Hox genes during the process, nor the mechanism that controls cell polarity.

Hereby we present a dynamical model of the molecular network that controls the competence, fate determination, and polarity of VPCs. The model was constructed by integrating the experimental information available in the literature on the roles of the different molecular components of the Wnt, Ras, and NOTCH signaling pathways, as well as the molecules that regulate the interactions between these pathways. Our model is the first to include the Wnt signaling pathway, the relevant Hox genes, and the molecules that control cell fusion.

## 2. Methods

### 2.1. Molecular basis of the regulatory network

#### 2.1.1. Expression patterns

**Figure A1:**
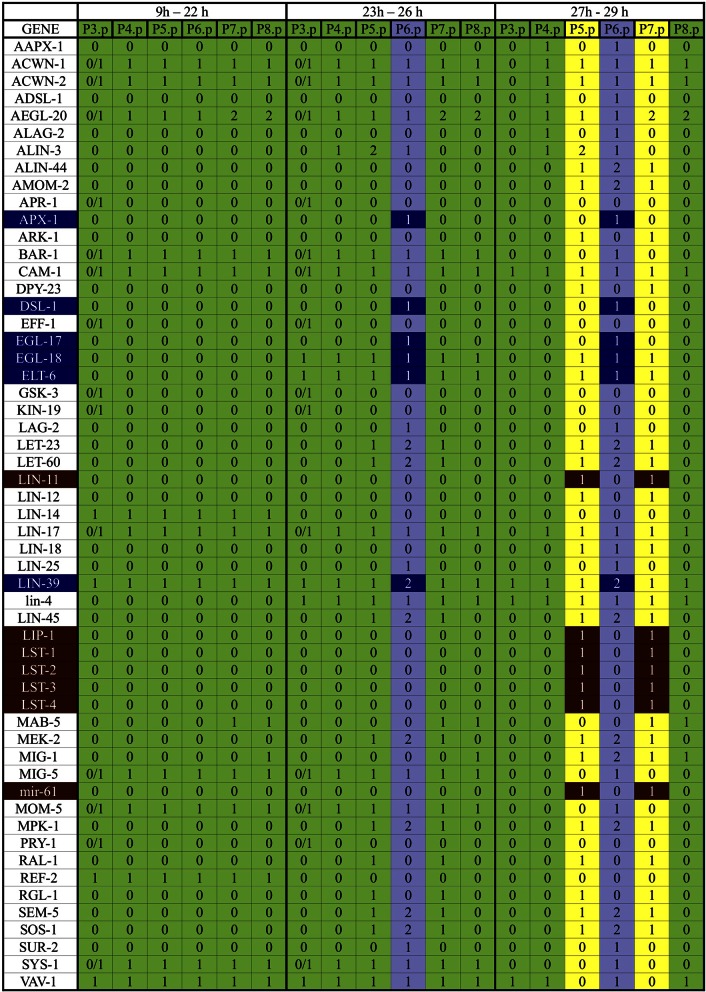
**Expected patterns of expression**. The patterns of expression colored in green correspond to the VPCs and the third fate, those in yellow correspond to the second fate, and those in light blue correspond to the first fate. The genes colored in dark blue are the first fate markers, and those colored in brown are the second fate markers. The proteins APH-1, APH-2, CEH-13, CEH-20, GAP-1, KSR-1, KSR-2, LAG-1, LIN-1, LIN-10, LIN-2, LIN-31, LIN-7, LIT-1, PEN-2, POP-1, SEL-12, SEL-8, SUP-17, UNC-62, VANG-1, WRM-1 are present in all six cells from 9h to 29h of development.

Before induction, VPCs have an active WNT signaling pathway, and they are characterized by a moderate LIN-39 activity and the presence of *lin-4*. First fate cells secrete DSL-1, and present APX-1, high levels of active LIN-39, MPK-1, and EGL-17. In the second fate cells the transcriptional complex CSL—formed by LIN-12, SEL-8, and LAG-1—is active, and the lateral signaling targets LIP-1 and LIN-11 are present. Finally, the third fate cells have the same pattern of activation as a VPC. A comprehensive list of the observed patterns of activation is included as Figure A1, which includes the patterns of expression that have been reported for the VPCs at different stages.

#### 2.1.2. Extracellular signaling

All vulval precursor cells are equivalent before induction. If a VPC is experimentally removed, another one –usually the nearest neighbor– acquires the fate that would correspond to the ablated cell in the wild type. Also, if all VPCs except P3.p are ablated, P3.p acquires the first fate (Sternberg and Horvitz, 1986). These results suggest that the extracellular concentration of three types of ligands, namely WNT, DSL, and EGF, determine the fate of VPCs, while the concentration of these ligands are determined by gradients determined by the relative positions of the VPCs and the AC (Sternberg and Horvitz, 1986; Wang and Sternberg, 1999).

#### 2.1.3. Formation of the vulval precursor cells

During larval phases L1 and L2, before induction, canonical RTK/Ras/MAPK and Wnt signaling maintain the competence of VPCs (Myers and Greenwald, 2007). The presence of the Hox gene *lin-39*, together with the absence of the Hox genes *mab-5* and *ceh-13*, are necessary for the formation and competence of the VPCs. Importantly, the expression of *mab-5* and *ceh-13* act as a boundary for the vulval equivalence group. As a result of the activation of Wnt and RTK/Ras/MAPK signaling cascades, the VPCs express LIN-39. This gene, together with its cofactors CEH-20 and UNC-62, activates the expression of *ref-2*, which inhibits the expression of the fusogen EFF-1. The posterior VPCs P7.p and P8.p express MAB-5, another Hox gene that activates the expression of *ref-2*. As a result, Wnt signaling mutants have a small penetrant effect on the posterior VPCs (Alper and Kenyon, 2002; Shemer and Podbilewicz, 2002; Shemer et al., 2004; Alper and Podbilewicz, 2008). During L1 and L2, the gradients of the Wnt ligands EGL-20 and CWN-1 are the most important factors controlling cell fusion. Moreover, these ligands may also be necessary for VPC competence, in a LIN-39-independent mechanism (Pénigault and Félix, 2011b). Finally, their anterior to posterior gradients reach a critical concentration near the VPC P3.p, with the result that P3.p fuse with hyp7 in half of the organisms (Green et al., 2008; Pénigault and Félix, 2011a).

#### 2.1.4. The canonical RTK/Ras/MAPK signaling cascade

The AC secretes LIN-3 (Hill and Sternberg, 1992), which binds to the receptors LET-23, LIN-2, LIN-7, and LIN-10, forming a agonist/receptor complex that localizes at the correct basso-lateral membrane (Simske et al., 1996; Kaech et al., 1998). When LIN-3 binds to LET-23, the receptor dimerizes and phosphorylates its C-terminal region exposing phospho-tyrosine residues that serve as docking sites for SEM-5. SEM-5 then recruits SOS-1 to activate LET-60, while GAP-1 directly inhibits LET-60 function. GTP-bound LET-60 binds to LIN-45 activating it, LIN-45 then binds to KSR-1 and KSR-2. The LIN-45/KSR-1/KSR-2 complex phosphorylates and activates MEK-2, which in turn phosphorylates MPK-1, which becomes active. MPK-1 then moves to the nucleus, where it phosphorylates target proteins, many of which are transcription factors like LIN-1, LIN-31, and LIN-39. Phosphorylated LIN-1 and LIN-31 are not able to form a complex that binds to the promoter –PJW-5– and inhibits the expression of *lin-39*. Instead, phosphorylated LIN-1 and LIN-31 activate the expression of LIN-39. Finally, phosphorylated LIN-39 activates its own expression (Tan et al., 1998; Sundaram, 2006; Wagmaister et al., 2006a), and the transcription of *lin-12* (Takács-Vellai et al., 2007).

#### 2.1.5. The canonical Wnt cascade

There are several Wnt ligands, CWN-1 and EGL-20 with penetrant phenotypes, and LIN-44, MOM-2, and CWN-2, with weak phenotypes. Also, there are several members of the Frizzled family of Wnt receptors, of which LIN-17, MIG-1, and MOM-5, are the most important during the vulva formation (Gleason et al., 2006). A Wnt ligand binds to a Frizzled-family Wnt receptor, and this membrane complex binds MIG-5 and APR-1. APR-1 forms a complex with KIN-19, GSK-3, and PRY-1. This complex marks the β-catenins BAR-1, WRM-1, and SYS-1 for ubiquitination and degradation. Also, when APR-1 is bound to the Frizzled receptor the concentration of BAR-1 increases. BAR-1 forms a complex with POP-1 (TCF), and activates the transcription of *lin-39* (Eisenmann, 2005; Wagmaister et al., 2006b).

#### 2.1.6. Determination of the first fate

Twenty-five hours after birth, P6.p responds to the EGF signal LIN-3 activating the canonical RTK-Ras-MAPK cascade, which strongly activates the expression and activity of LIN-39 that in turn activates the transcription of *egl-17* (Cui and Han, 2003). Both LIN-39 and *egl-17* are markers of the first cell fate. The increased expression of LIN-39 in P6.p during this stage depends on SUR-2 and LIN-25, subunits of the Mediator complex (Nilsson et al., 1998; Wagmaister et al., 2006b). The expression of the main components of the lateral signal emitted by the first cell fate, namely the DSL ligands LAG-2, DSL-1, and APX-1 is also SUR-2 dependent in the VPC P6.p. While the ligands LAG-2 and APX-1 are localized on the membrane of P6.p, DSL-1 is secreted (Chen and Greenwald, 2004). During the determination of the first fate, LIN-39 acts with its cofactor CEH-20 to activate the transcription of *elt-5/egl-18* and *elt-6*. ELT-5 and ELT-6 inhibit *eff-1* expression inhibiting the fusion of first fate cells with hyp7 (Koh et al., 2002, 2004; Alper and Podbilewicz, 2008).

#### 2.1.7. Determination of the second fate

NOTCH signaling is a key element for the determination of the second cell fate. Before the larval phase L3, LIN-14 inhibits LIN-12, preventing the second fate determination. Later, *lin-4* RNA concentration increases, inhibiting the expression of *lin-14* by binding to its mRNA and targeting it for degradation (Li and Greenwald, 2010). Twenty-eight hours after eclosion, P5.p and P7.p cells respond to the lateral signal expressed by P6.p, due to the activation of the NOTCH signaling cascade by the DSL ligand (Chen and Greenwald, 2004). The signal activates LIN-12 (NOTCH), which undergoes a SUP-17 mediated cleavage at the extracellular site 2, and then is cleaved again at the transmembrane site 3 mediated by the γ-secretase protease complex conformed by SEL-12 (HOP-1, the catalytic subunit), APH-1, APH-2, and PEN-2. The resulting intracellular domain of LIN-12 is translocated to the nucleus where it binds to LAG-1 (CSL) and SEL-8, forming a complex that activates the transcription of the target genes *ark-1, lip-1, dpy-23, lst-1, lst-2, lst-3, lst-4*, and *lin-11*, among others. Importantly, LIP-1 and LIN-11 are markers of the second cell fate (Greenwald, 2005). Cells that acquire the second fate inhibit the expression of *eff-1*, very likely mediated by the LAG-1/SEL-8 complex represented by the CSL node since some regulatory regions of *eff-1* contain candidate LAG-1/CSL binding sites and NOTCH signaling inhibits the fusogenic function of *eff-1* during the formation of the digestive tract of *C. elegans* (Rasmussen et al., 2008). Finally, the second fate has at least two positive feedback circuits. First, LIN-12 activates the LAG-1/SEL-8 complex which in turn activates *lin-12* transcription (Wilkinson et al., 1994). And second, LIN-12 activates *mir-61* transcription, which causes VAV-1 down-regulation, and as a result promotes *lin-12* activity (Yoo and Greenwald, 2005).

There is another mechanism involved in the determination of the second fate. It has been reported that a diminished LIN-3/EGF signal directly promotes the second fate (Sternberg and Horvitz, 1986, 1989; Katz et al., 1995, 1996). As a result, a model has been proposed (Zand et al., 2011) where a small concentration of LIN-3/EGF causes LET-60/Ras to activate RGL-1, which then activates RAL-1/RalGEF instead of Ras activating LIN-45/Raf, and thus inhibiting the first fate and indirectly promoting the second fate. The authors of the model mentioned above also proposed that RAL-1 may directly activate NOTCH signaling.

#### 2.1.8. The first and second fates inhibit each other

The SUR-2 dependent LIN-12 endocytosis and/or degradation is activated in P6.p (Shaye and Greenwald, 2002), thus promoting the first cell fate. In turn, the lateral signal targets inhibit RTK-Ras-MAPK signaling in the VPCs that adopt the second fate. Specifically, LIP-1 inactivates MPK-1 (Berset et al., 2001), while ARK-1 inhibits LET-23 in a SEM-5 dependent mechanism (Hopper et al., 2000). Also, the lateral signal targets *lst-1, lst-2, lst-3, lst-4*, and *dpy-23*, inhibit first fate determination in P5.p and P7.p. The loss-of-function mutants of those same targets have phenotypes characterized by the ectopic expression of *egl-17* (Chen and Greenwald, 2004).

#### 2.1.9. The gradients of different WNT ligands determine the polarity of the VPCs

The ligand EGL-20 binds to the receptors CAM-1 and VANG-1 to establish the ground polarity of the VPCs. EGL-20 is secreted by the cells near the posterior end of the worm, so that its concentration is higher in the posterior part of all VPCs (Green et al., 2008). During larval phase L3 the AC secretes the ligands LIN-44 and MOM-2, which bind to the receptors LIN-17 and LIN-18. After these two events, the concentration of polarizing WNT ligands is higher in P5.pp than P5.pa, and higher in P7.pa than in P7.pp. In the cells with a higher concentration of polarizing WNT ligands, the β-catenins BAR-1, WRM-1, and SYS-1 are not degraded, WRM-1 forms a complex with LIT-1 and POP-1 that moves out of the nucleus (Lo et al., 2004), and thus the ratio of SYS-1 to POP-1 is sufficiently large to activate the transcription of some target genes (Eisenmann, 2005; Green et al., 2008).

### 2.2. The regulatory network as a discrete dynamical system

Boolean networks constitute the simplest approach for modeling the dynamics of regulatory networks. These networks consist of a set of nodes, usually representing genes or proteins, each of which may attain one of only two possible states; namely, 0 if the node is inactive and 1 if the node is active. The state of activation of the *i* th node is represented by *x*_*i*_, and is updated in discrete time steps according to a Boolean function *F*_*i*_ such that *x*_*i*_(*t* + 1) = *F*_*i*_[*x*_1_(*t*), *x*_2_(*t*), …, *x*_*n*_(*t*)], where [*x*_1_(*t*), *x*_2_(*t*), …, *x*_*n*_(*t*)] is the state of the regulators of *x*_*i*_ at time *t*. In the simplest case the functions *F*_1_, *F*_2_, …, *F*_*n*_ are solved simultaneously, which is known as a the synchronous approach. In such case, the dynamics of the Boolean network is deterministic, and for any given initial state the network reaches either a fixed-point or a cyclic state. The set of all these asymptotic behaviors is known as the attractors of such network. Kauffman (1969, 1993) proposed that the attractors of Boolean networks represent the experimentally observed gene expression profiles that characterize different cell types.

We developed a discrete dynamical system of the network that controls fate determination, function, and polarity of the VPCs based on the molecular basis of the regulatory network. We used a generalization of a Kauffman network (Kauffman, 1969), where nodes may attain two or more states of activation (Mendoza, 2006; Sánchez et al., 2008; Schlatter et al., 2009; Franke et al., 2010). Specifically, in our model there are seven nodes with four possible levels of activation –LIN-3^*^, LET-23, SEM-5, SOS-1, LET-60, LIN-45, and MEK-2–. The reason is that the VPCs P3.p, P8.p, and P9.p, which acquire the third fate, have no Ras activity (i.e., a level of 0); the VPCs P5.p and P7.p usually have a moderate level of Ras signaling which is sufficient to determinate the second fate (i.e., a level of 1); P6.p is characterized by a high level of Ras signaling (i.e., a level of 2), which is sufficient to determinate the first fate, but only in the absence of negative regulators like GAP-1, ARK-1 and LIP-1; and finally, in some experiments with worms that have more then one ACs, the level of Ras signaling is high enough to overcome the effects of the negative regulators (i.e., a level of 3). For the genes lin-3, lin-23 and lin-60, the phenotypic effects of some mutant alleles correspond to the different levels of activity described above. Moreover, five nodes need three levels of activation. MPK-1, LIN-39, and LIN-39a, which are at the end of the Ras signaling cascade or downstream from it, but they have no inhibitors to overcome, so only the levels 0, 1, and 2 are needed. Then, PJW-5 is a promoter of lin-39, which is completely inactivated by unphosphorylated LIN-1 (level 0), the loss of function of LIN-1 causes a moderate level of activity in PJW5 (level 1), and phosphorylated LIN-1 and LIN-31 completely activates PJW5 (level 2). Finally, EGL-20^*^ also requires three levels, the highest (level 2) is enough to activate MAB-5, a medium level (level 1) is enough to polarize the VPCs and activate POP-1, and its absence (level 0) is needed to allow VPCs to fuse with hyp7. For the rest of the nodes, the experimental evidence report either a full gain or total loss of function, and therefore only 2 levels of activation are necessary. The rules determining the state of activation of each node as a function of their regulatory inputs, as well as the references used to infer such rules are shown in the Table A1.

It is important to note that the model includes two kinds of nodes which activity does not change during the simulation, and hence act as parameters of the system. First, the proteins that represent the extracellular molecular environment; namely, LIN-3^*^, MOM-2^*^, CWN-2^*^, LIN-44^*^, CWN-1^*^, EGL-20^*^, LAG-2^*^, DSL-1^*^, and APX-1^*^. And second, nodes that represent genes whose expression and activity is the same in all the VPCs and the three fates; namely, LIN-2, LIN-7, LIN-10, GAP-1, KSR-1, KSR-2, VANG-1, LIT-1, CEH-13, CEH-20, UNC-62, lin-4, SUP-17, APH-1, PEN-2, LIN-1, LIN-31, and SEL-8. It is important to mention that throughout our study we modeled only one VPC in several possible environments, thus avoiding an excessive complexity of the model.

We obtained all the attractors of the discrete dynamical system with the use of GINsim (Gonzalez et al., 2006). The reader may find the GINsim implementation of our model as the Supplementary Materials *vpcwt23h.ginml*. Besides the attractors of the wild type model, we obtained the attractors for all possible single loss- and gain-of-function mutations, included as Supplementary Materials *mutants.xls*. Furthermore, we also obtained the attractors of the network after systematically removing one interaction at a time; such results are included as Supplementary Materials *interactions.xls*. Finally, we simulated the processes of fate determination, and the transitions from one cell type to another with the use of a script (Supplementary Materials vpc.py).

## 3. Results

### 3.1. The regulatory network

**Figure 2:**
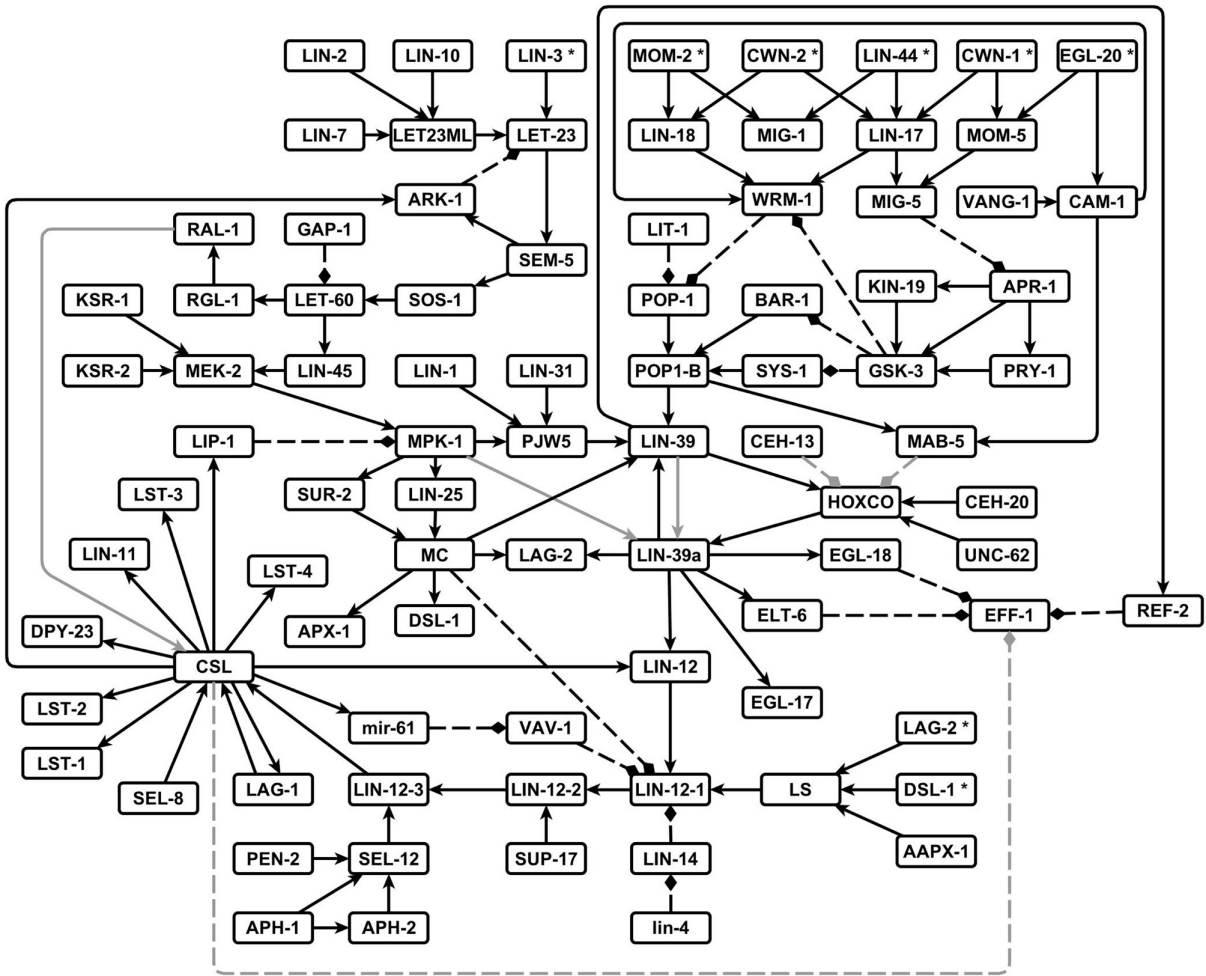
**The network that controls the VPC fate determination and cell fusion in *C. elegans***. Pointed arrows are positive regulatory interactions, and dashed blunt arrows are negative regulatory interactions. The interactions predicted by others and supported by our model are shown in gray.

We were able to reconstruct the regulatory network that controls the VPC fate determination and cell fusion, made of 88 nodes and 126 regulatory interactions (Figure 2). Most interactions were inferred from the experimental data mentioned in the methodology. Four interactions, however, are predictions that are supported by our modeling effort; namely, (1) RAL-1 activates the protein complex that allows the lateral signal targets to be expressed, (2) the self activation of LIN-39 transcription requires LIN-39 to be phosphorylated by MPK-1, (3) the complex formed by LAG-1, LIN-12 and SEL-8 inhibits the transcription of *eff-1* in the second fate cells, and (4) the Hox factors MAB-5 and CEH-13 inhibit vulval fate determination by rendering the cofactors UNC-62 and CEH-20 unavailable to LIN-39.

In our model, three or four levels of activation are necessary in some nodes (see Methods) to simulate the effect of additional ACs, with the result of a higher concentration of LIN-3, as well as to describe the reported effects of different mutant alleles.

### 3.2. Stationary patterns of activation

**Figure 3:**
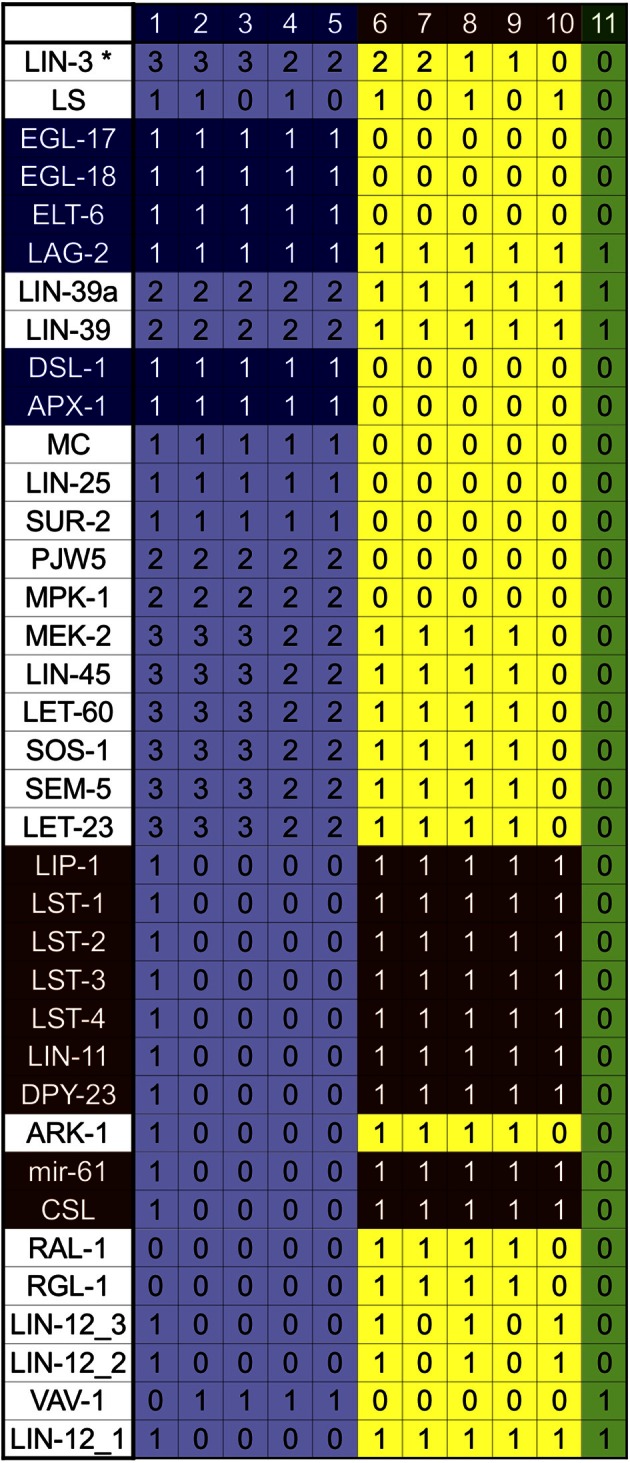
**Attractors of our model of the wild type VPC**. The patterns of expression colored in green correspond to the VPCs and the third fate, those in yellow correspond to the second fate, and those in light blue correspond to the first fate. The genes colored in dark blue are the first fate markers, and those colored in brown are the second fate markers. The molecules CWN-1^*^, CWN-2^*^, EGL-20^*^, LIN-44^*^, MOM-2^*^, APH-1, APH-2, BAR-1, CAM-1, CEH-20, GAP-1, HCO, KSR-1, KSR-2, LAG-1, LET-23ML, LIN-1, LIN-10, LIN-12, LIN-17, LIN-18, LIN-2, LIN-31, lin-4, LIN-7, LIT-1, MIG-1, MIG-5, MOM-5, PEN-2, POP-1, POP-1b, REF-2, SEL-12, SEL-8, SUP-17, SYS-1, UNC-62, VANG-1, and WRM-1 are present and active at level 1, in all attractors. The molecules APR-1, CEH-13, EFF-1, GSK-3, KIN-19, LIN-14, MAB-5, and PRY-1 are not active in any of the attractors.

The analysis of the dynamical behavior shows that the network has 11 fixed point attractors (Figure 3), all of which can be interpreted as the stable patterns of molecular activation possible for different vulval cells. According to their biological interpretation, these attractors can be grouped into three categories: first fate, second fate, and third fate/VPC.

The first group contains five attractors corresponding to the first cell fate. Attractors in this group are characterized by a high level of activation in the Ras/MAPK signaling pathway, a high level of activation of LIN-39, and the expression of *apx-1, dsl-1, egl-18, elt-6*, and *egl-17*, which is used as a first fate molecular marker. Attractors 1–3 present the highest possible level of extracellular LIN-3. A high level of inductive signaling is enough to determine the first fate, even in the presence of NOTCH signaling, as observed in attractor 1. This particular pattern of activation recovers the expression observed in Sternberg and Horvitz (1989) and Wang and Sternberg (1999), where VPCs acquire the second fate, but they respond to LIN-3 by becoming first fate cells that express *egl-17*, and whose granddaughters divide transversely. Attractor 4 represents a case where a VPC acquires the first fate with an extracellular LIN-3 concentration as found in P6.p in the wild type, such pattern has already been reported Sternberg and Horvitz (1989). And finally, attractor 5 corresponds to the pattern of expression reported for P6.p after acquiring the first fate. The determination of the first fate in P6.p occurs in an extracellular environment with a high concentration on LIN-3 and no lateral signaling.

The second group, comprised of attractors 6–10, correspond to the second cell fate. These attractors are characterized by the activity of the LAG-1/SEL-8 complex, represented by the CSL node, as well as the expression of the lateral signal targets *lst-1, lst-2, lst-3, lst-4, mir-61, dpy-23, lin-11*, and *lip-1*. Importantly, *lin-11* and *lip-1* are routinely used as second fate markers. Attractor 6 has both a high level of extracellular LIN-3 and an active lateral signaling, as reported in Sternberg and Horvitz (1989). Attractor 7 presents a high level of the inductive signaling, together with active RGL-1 and RAL-1 but no lateral signaling. This pattern has not been reported in the literature and therefore constitutes a prediction of the model. Attractor 8 fits the pattern of activation and the extracellular conditions for P5.p and P7.p, which acquire the second fate in the wild type. These conditions, include a small extracellular concentration of LIN-3 and active lateral signaling. Attractor 9 has a low inductive signaling, active RGL-1 and RAL-1, and no lateral signaling. This kind of pattern has been proposed so as to explain the determination of the second fate in an extracellular environment lacking lateral signaling (Zand et al., 2011). Finally, attractor 10 has an active lateral signal, but no inductive signal, as reported in Wang and Sternberg (1999).

The third group contains only one attractor—number 11—which represents the third fate. This attractor is characterized by a low level of LIN-39, and well as the activation of *lin-4, lin-12*, and *ref-2*. Importantly, the very same pattern of activation is found in the VPCs after the L3 molt but before induction.

In our model we use nine parameters to simulate the extracellular environment, denoted with an asterisk (APX-1^*^, CWN-1^*^, CWN-2^*^, DSL-1^*^, EGL-20^*^, LAG-2^*^, LIN-3^*^, LIN-44^*^, and MOM-2^*^) in all the attractors described above. Importantly, the Wnt ligands (CWN-1^*^, CWN-2^*^, EGL-20^*^, LIN-44^*^, and MOM-2^*^) were set to an active state that prevents cell fusion. To model the fusion of P3.p with hyp7, these five parameters are set to 0. In this specific case, the model has only one attractor, which corresponds to the fusion fate, evidenced by the active state of the fusogen EFF-1.

### 3.3. The differentiation process

**Figure 4:**
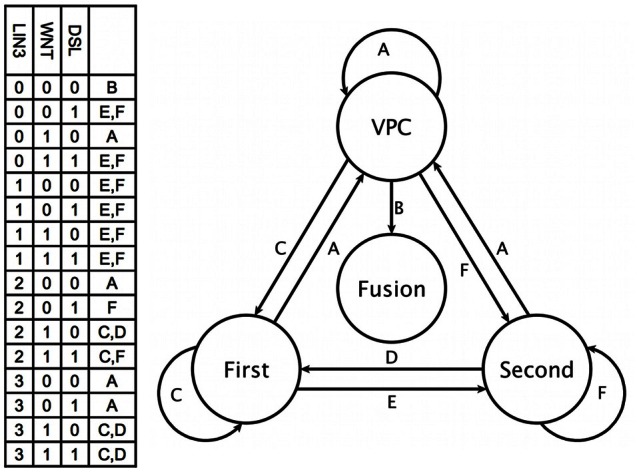
**Fate determination**. The combination of key environmental signals (table on the left) determine the cell type (circles at the right) in our model. Therefore, a change to a new combination of such signals (labels above the arrows) results in the eventual differentiation of the cell (arrows).

**Figure A2:**
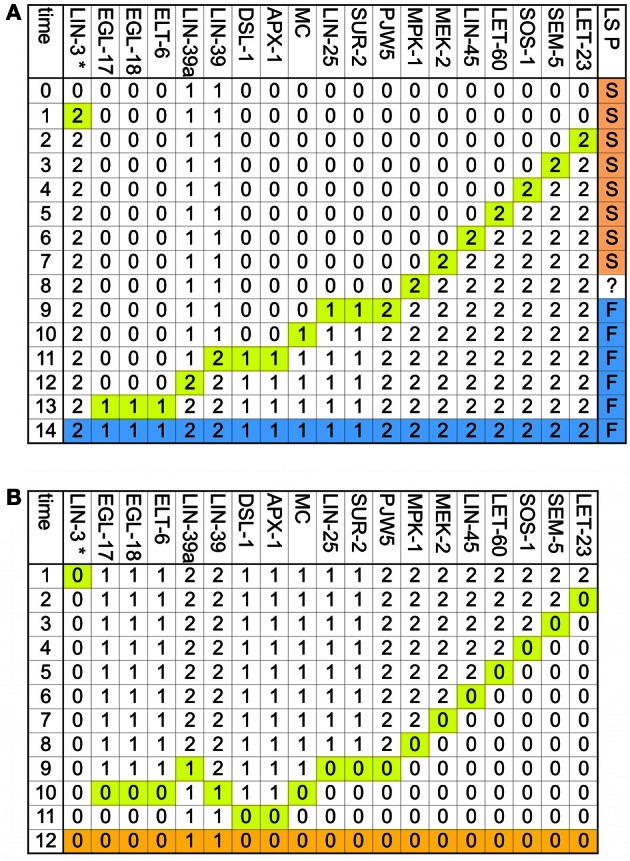
**The determination of the first fate is reversible**. Only the nodes whose activity changes dynamically are included, and the time at which they change is highlighted in yellow. **(A)** Transition from VPC to the first fate. The final pattern, which corresponds to the first fate is colored in blue. Also included in this figure, as the LS P column, are the effects of the addition of DSL ligands to the environment at different stages of first fate determination, if the DSL ligands are added before the eighth step, the VPC acquires the second fate instead of the first, if the environment changes at the eighth step, the VPC acquires a fate that cycles between an incomplete first fate and an incomplete second fate, if the DSL ligands are added to the environment after the eight step, the VPC acquires the first fate. **(B)** Transition from the first fate to VPC. The final pattern, which corresponds to the VPC is colored in orange.

**Figure A3:**
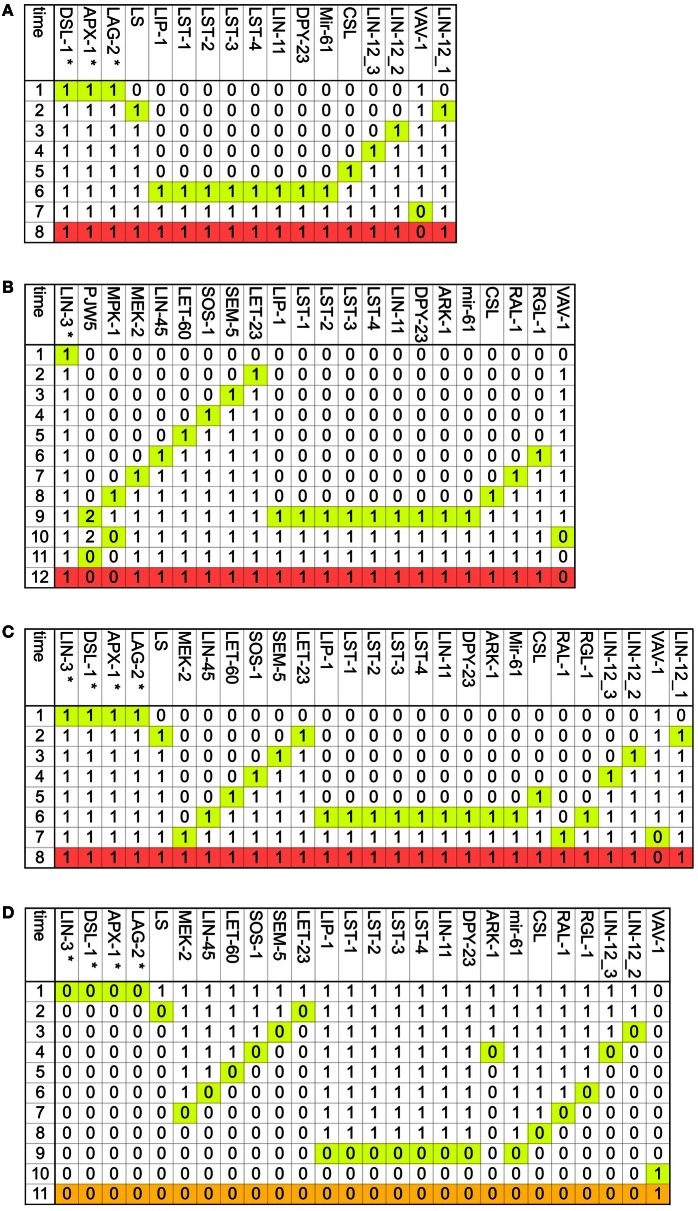
**The determination of the second fate is reversible**. Only the nodes whose activity changes dynamically are included, the time at which they change is highlighted in yellow and the pattern, which corresponds to the second fate is colored in salmon. **(A)** Transition from VPC to the second fate in an environment with DSL ligands but no LIN-3. **(B)** Transition from VPC to the second fate in an environment with a moderate concentration of LIN-3, but no DSL ligands. **(C)** Transition from VPC to the second fate in an environment with DSL ligands and a moderate concentration of LIN-3. **(D)** Transition from the second fate to a VPC. The final pattern, which corresponds to the VPC is colored in orange.

The dynamical modeling of the network is able to describe the capacity of the VPC cells to acquire the first, second, and fusion fates (Figure 4). We simulated the determination of the first fate starting from a VPC (Figure A2A). While the pattern of a VPC is a stationary state, the sudden activation of LIN-3 to its highest level, which simulates the arrival of a high inductive signal from the AC, originates a cascade of activation that activates the Ras/MAPK signaling cascade, inducing the activation of LIN-39, EGL-17, ELT-6, and EGL-18. Another effect of the activation of Ras signaling pathway is the endocytosis of LIN-12 by the Mediator complex. While the VPC and first state attractors are stationary, the transition of the former to the latter is reversible (Figure A2B). Indeed, the disappearance of the inductive signal, simulated by turning LIN-3 from its highest to its lowest value of activation, reverses the changes described above. Thus, our model shows that if a first fate cell is moved into an environment with WNT ligands but lacking LIN-3, such cell becomes a VPC; a similar effect has been observed experimentally (Euling and Ambros, 1996; Wang and Sternberg, 1999). The second fate may be acquired by a VPC in an environment containing DSL ligands (Figure A3A), as described experimentally (Sternberg and Horvitz, 1989; Wang and Sternberg, 1999; Chen and Greenwald, 2004). The presence of the lateral signal activates the NOTCH signaling, which leads to the activation of the lateral signal targets (Yoo et al., 2004). In our model, a VPC also acquires the second fate in an environment with a moderate concentration of LIN-3, which is known to occur (Katz et al., 1995, 1996; Hoyos et al., 2011; Zand et al., 2011). We propose that this effect may happen through the direct activation of the CSL transcriptional complex by RAL-1 (Figure A3B). Also, in an environment with both DSL ligands and a moderate concentration of LIN-3 the cells P5.p and P6.p acquire the second fate (Sternberg and Horvitz, 1989), a behavior that is also captured by our model (Figure A3C). In this case the lateral signal targets are activated by the lateral signal before the activation of RAL-1. Finally, as with the first fate, the second fate also shows reversibility of differentiation (Figure A3D). When a second fate cell is moved into an environment with WNT ligands but lacking LIN-3 and lateral signal, the cell would de-differentiate and become a VPC, reflecting an experimentally observed behavior (Euling and Ambros, 1996).

**Figure A4:**
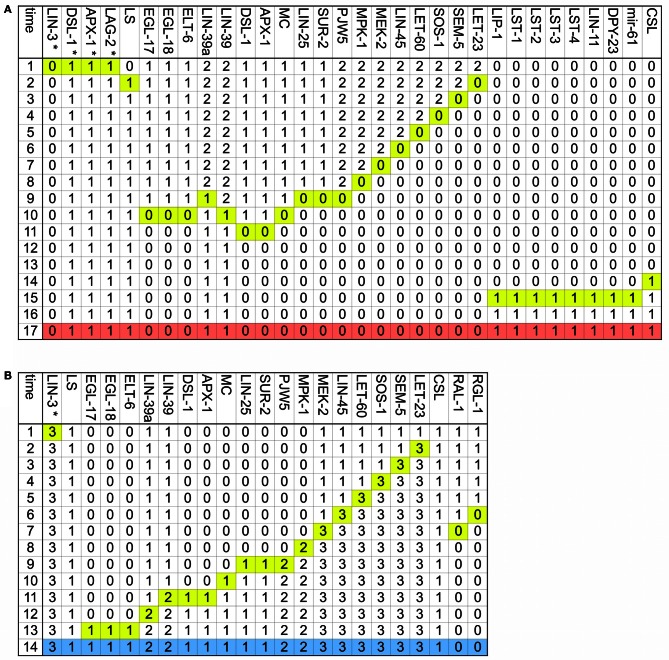
**The two main vulval fates may transdifferentiate**. Only the nodes whose activity changes dynamically are included, and the time at which they change is highlighted in yellow. **(A)** Transition from the first fate to the second fate. The final pattern, which corresponds to the second fate is colored in salmon. **(B)** Transition from the second fate to the first fate. The final pattern, which corresponds to the first fate is colored in blue.

Our model predicts that a first fate cell in an environment with a lateral signal but no inductive signal would acquire the second fate (Figure A4A). Conversely, when a second fate cell is moved into an environment with DSL ligands and a very high concentration of LIN-3 (Figure A4B). The Ras/MAPK signaling activates the first fate markers, while the lateral signal targets remain active, reflecting an experimentally observed behavior (Sternberg and Horvitz, 1989; Wang and Sternberg, 1999).

**Figure A5:**
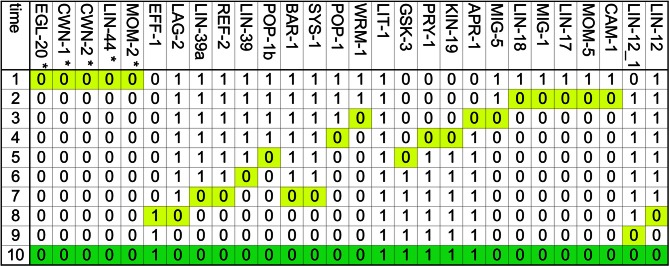
**Transition from VPC to the fusion fate**. Only the nodes whose activity changes dynamically are included, and the time at which they change is highlighted in yellow. The final pattern, which corresponds to the fusion fate is colored in green.

Finally, it is known that in half of all worms P3.p fuses with hyp7 at about twelve hours after birth. This is determined by the concentration of EGL-20 and CWN-1, which are the only Wnt ligands reaching the cell at this stage of development (Myers and Greenwald, 2007; Pénigault and Félix, 2011a,b). In order to simulate the transition to the fusion fate it was necessary to change the parameters of the simulation, so as to reproduce an environment with no Wnt, LIN-3 or lateral signaling. The resulting dynamics is presented in Figure A5, showing that an environment lacking a sufficient concentration of WNT, DSL ligands, and LIN-3, the fusogen EFF-1 is activated and therefore the VPC acquires the fusion fate.

### 3.4. Simulation of mutants

Experimentally, mutations on genes that control the formation of the vulva in *C. elegans* may cause the following phenotypes: (1) a vulva-less hermaphrodite (Vul), (2) an hermaphrodite with multiple vulvae (Muv), (3) a worm with two incomplete vulvae (Biv), (4) a worm defective in egg laying (Egl), (5) a worm with at least one protrusive vulva (Pvl), and (6) a fertile individual whose eggs hatch inside the worm (Bag).

We simulated the effect of mutations that cause each molecule to stay at a fixed state of activation (Table A2), and it was possible to assign the development of Vul, Muv, and Egl phenotypes (Table 1). Accordingly, we compared the simulated mutants against the reported mutant phenotypes (Table A3). Importantly, the model was able to describe most of the reported mutants, namely, 15 of 19 type Vul, 11 of 17 Muv, 19 of 32 Egl, 8 of 8 Biv, and 24 out of 26 wild types. Only a few mutants where incorrectly classified, specifically 3 mutants as Vul, 1 as Muv, 3 as Egl, and 23 as wild type. Most of the discrepancies with the reported phenotypes are due to three reasons. First, considering RAL-1 activity as sufficient to determinate the second fate causes many NOTCH mutants to lose their effect. Second, some of the mutants have an effect at a later stage of vulval development not included in our model. And third, some mutants have an effect on less than half of the worms affected, and we could not include such effects due to the deterministic nature of our model.

**Table 1:**
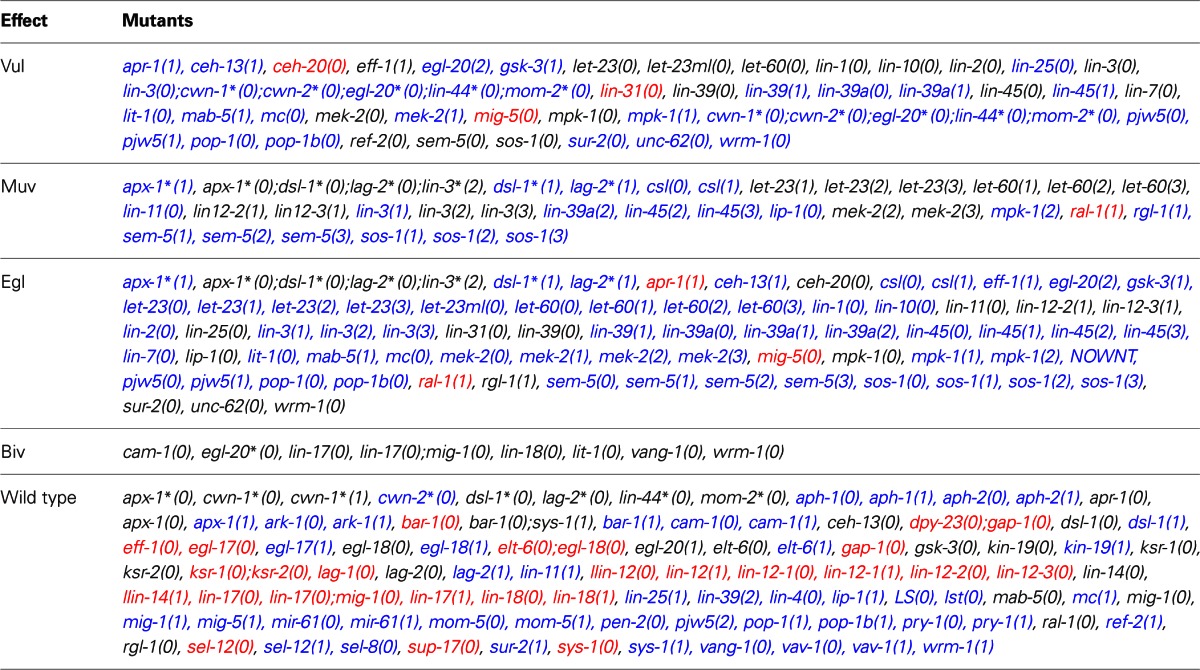
**Simulation of mutants and their phenotypic effect**.

*Mutants in black have a phenotype that reproduce what is reported in the literature; mutants in red have a simulated effect that differs from what is reported in the literature; and mutants in blue are predictions of our model*.

Additionally, the model also allows for the appearance of the Biv phenotype. Specifically, if the presence of second fate cells is possible, the Biv phenotypes arises if the basal polarity of the vulval precursor cells is lost (loss of function mutants for the genes egl-20^*^, cam-1, vang-1), or the re-polarization by the ACs is affected in mutants with combinations of the loss of function of the genes *lin-44, mom-2, lin-17, lin-18, wrm-1 or sys-1*.

### 3.5. Effect of removing a single interaction at a time

Removing one of 35, out of a total of 115 interactions, cause the model to recover an incorrect dynamical behavior. This is shown in Table A4, which incorporates a partial robustness analysis. These 35 interactions comprise those that are part of the Ras signaling cascade, those that are downstream of the Wnt signaling cascade, those that are essential for ref-2 to inhibit cell fusion, and those which activate the second fate markers.

## 4. Discussion

### 4.1. Implications of the model on some selected molecular mechanisms

Our model is able to recover the second fate in an extracellular environment without DSL ligands and with a moderate concentration of LIN-3 (Figures 4, A3B). While this process does not fit the classical description outlined in the introduction, it has been observed experimentally nonetheless (Katz et al., 1995, 1996). One possible explanation is that the low extracellular concentration of LIN-3 activates Ras signaling in such a way that LET-60 activates RGL-1 instead of LIN-45; RGL-1 then activates RAL-1 which may then cause the determination of the second fate (Zand et al., 2011). Our model supports such untested hypothesis, because of the high threshold necessary for extracellular LIN-3 to activate the transcription of *dsl-1*. While the mechanism for the pro-second fate effect of RAL-1 is not yet clear, we include in our network a regulatory interaction from RAL-1 to CSL; not including this interaction results in the VPC acquiring the third fate instead of the second.

The up-regulation of *lin-39* by Ras requires basal levels of LIN-39 (Maloof and Kenyon, 1998). The activity of *lin-39* is necessary for the formation of the VPCs, the determination of the fates and cell fusion control. Experimental evidence supporting that LIN-39 is a target for MAP kinase *in vitro*, and that LIN-39 up-regulates its own transcription has been published (Tan et al., 1998; Wagmaister et al., 2006b). To include such information in our model, we propose that the self activation of *lin-39* is dependent on MPK-1 by adding an interaction from MPK-1 to LIN-39a. Notably, the removal of such interaction does not affect the fixed point attractors or the cell fate transitions.

In pm8 epithelial cells, which are part of the pharynx of *C. elegans*, Notch signaling inhibits the expression of *eff-1* and the regulatory region of *eff-1* contains candidate LAG-1/CSL binding sites (Rasmussen et al., 2008). While it is still necessary to test experimentally the control of cell fusion by Notch signaling in second fate vulval cells, in our model CSL activity is enough to inhibit *eff-1*. If we remove the regulation of EFF-1 by CSL, and then set a VPC with an environment containing DSL ligands and lacking WNT ligands, the VPCs acquire the fusion fate instead of the second fate.

MAB-5 is usually expressed in P7.p and P8.p. Ectopic expression of MAB-5 in all VPCs reduces their sensitivity to the inductive signal, and in *mab-5(lf)* mutants, the 3 posterior VPCs are all very likely to acquire the first fate (Clandinin et al., 1997). In *ceh-13(lf)* hermaphrodites several anterior and posterior Pn.p cells remain unfused, and *mab-5(gf)*;*ceh-13(lf)* mutants are almost wild type, suggesting that mab-5 may substitute for CEH-13 to control the fusion of Pn.p cells (Tihanyi et al., 2010). Based on this information, in our model we propose that the presence of either *mab-5* or *ceh-13* decrease the amount of cofactors available for *lin-39* activity. Not including either the MAB-5 to HOXCO or the CEH-13 to HOXCO interactions, would allow a VPC to acquire a vulval fate even in the presence of MAB-5 or CEH-13.

Another interesting property of our model is that the VPCs that acquire the third fate, do not fuse with hyp7 before dividing because the concentration of Wnt ligands required for the proper specification of the first fate, which are secreted by the AC is high enough to prevent cell fusion. After proper first fate specification and the first VPC division, the concentration of Wnt ligands must drop bellow the critical concentration required to prevent cell fusion.

### 4.2. Limitations of the model and some possible improvements

Our model has 88 nodes some of which have up to 4 possible levels of activation, and the state space of the model is very large 4^7^ × 3^5^ × 2^76^ = 3.008194e + 29, exploring each possible initial state would take a very long time. The use of initial conditions that are biologically relevant, and the efficient algorithm used by GINsim allowed us to find all the fixed point attractors for all our different versions of the network. Simulating the loss of an interaction or a mutation produces a new network, thus, we decided to study only the most important paths that lead from one cellular fate to another one, using the known pattern of gene activity in the VPCs as a starting point.

We decided to incorporate the regulatory interaction from RAL-1 to CSL because it improves the whole dynamical behavior of the wild type model. While the absence of such interaction would result in the worsening of the wild type behavior, it would improve the dynamics describing the following mutants: *aph-1(0)*, *aph-2(0)*, *lag-1(0)*, *lin-12(0)*, *lin-14(1)*, *lin-4(0)*, *pen-2(0)*, *sel-12(0)*, *sel-8(0)*, and *sup-17(0)*. A possible solution to reconcile this misbehavior would be to postulate that RAL-1 somehow activates the expression of DSL-1. We will explore this possibility in the near future.

There are two general changes that might improve the model. First, the implementation of the network as a stochastic model. And second, the inclusion of events that occur after the first longitudinal division of the VPCs. All these changes will be explored separately.

## 5. Conclusion

Our model is the largest reconstruction to date of the molecular network controlling the specification of vulval precursor cells and cell fusion control in *Caenorhabditis elegans*. According to our model, the process of fate determination in the vulval precursor cells is reversible, at least until either the cells fuse with the ventral hypoderm or divide, and therefore the cell fates must be maintained by the presence of extracellular signals, in agreement with a previous hypothesis (Euling and Ambros, 1996). Furthermore, our model predicts that the trans-differentiation from the first to second fate and vice versa are possible (Figure A4).

Most previous models that describe vulval formation require a gradient in the inductive signal for proper fate specification, or alternatively, require the inductive signal reaching the first fate cells before the lateral signal (and the reverse order for second fate cells). Remarkably, our bottom-up approach of reconstruction of the network resulted in a model where either of the two previous mechanisms is sufficient for proper fate determination.

### Conflict of interest statement

The authors declare that the research was conducted in the absence of any commercial or financial relationships that could be construed as a potential conflict of interest.

## Figures and Tables

**Table A3:**
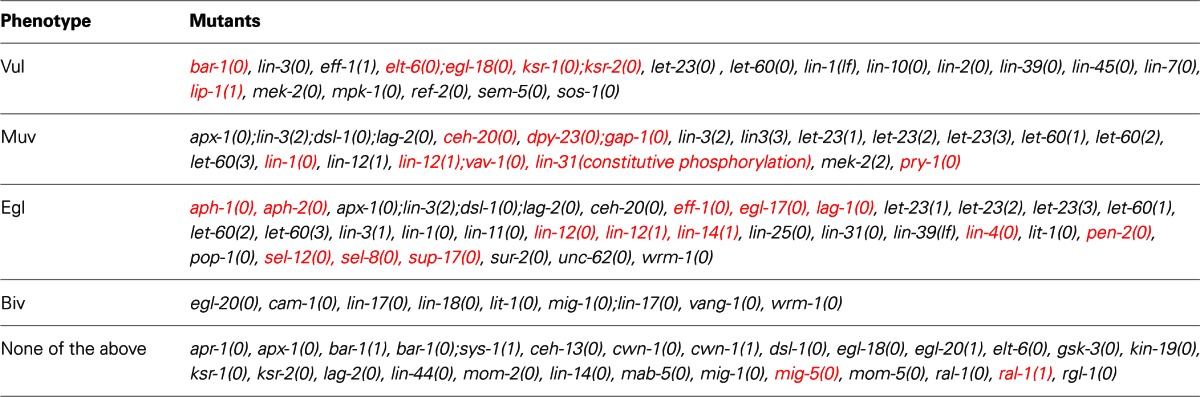
**Reported effect of different mutations**.

*The mutants in red were not completely reproduced by our model. In total, we found 102 relevant mutants, our model correctly reproduces the effect of 77: 15 out of 19 Vul phenotypes, 11 out of 17 Muv phenotypes, 19 out of 32 Egl phenotypes, 24 out of 26 wild types, and all 8 Biv phenotypes*.

## Appendix

**Table A1:** **The VPC cell network as a discrete dynamical system**.

**Rule**	**References**
APX-1^*^(t + 1) = APX-1^*^(t)	Chen and Greenwald, 2004
CWN-1^*^(t + 1) = CWN-1^*^(t)	Gleason et al., 2006; Pénigault and Félix, 2011b
CWN-2^*^(t + 1) = CWN-2^*^(t)	Gleason et al., 2006
DSL-1^*^(t + 1) = DSL-1^*^(t)	Chen and Greenwald, 2004
EGL-20^*^(t + 1) = EGL-20^*^(t)	Gleason et al., 2006; Pénigault and Félix, 2011b
LAG-2^*^(t + 1) = LAG-2^*^(t)	Chen and Greenwald, 2004
LIN-3^*^(t + 1) = LIN-3^*^(t)	Ferguson and Horvitz, 1985; Hill and Sternberg, 1992
LIN-44^*^(t + 1) = LIN-44^*^(t)	Green et al., 2008; Gleason et al., 2006
MOM-2^*^(t + 1) = MOM-2^*^(t)	Green et al., 2008; Gleason et al., 2006
APH-1(t + 1) = APH-1(t)	Francis et al., 2002; Goutte et al., 2002
APH-2(t + 1) = APH-1(t)	Goutte et al., 2000; Levitan et al., 2001; Goutte et al., 2002
APR-1(t + 1) = NOT (MIG-5(t))	Hoier et al., 2000; Gleason et al., 2002; Eisenmann, 2005
APX-1(t + 1) = MC(t)	Chen and Greenwald, 2004
ARK-1(t + 1) = CSL(t) AND SEM-5(t)	Yoo et al., 2004; Hopper et al., 2000
BAR-1(t + 1) = NOT (GSK-3(t))	Eisenmann et al., 1998; Gleason et al., 2002; Eisenmann, 2005
CAM-1(t + 1) = EGL-20^*^(t) AND VANG-1(t)	Green et al., 2008
CEH-13(t + 1) = CEH-13(t)	Bürglin and Ruvkun, 1993; Tihanyi et al., 2010
CEH-20(t + 1) = CEH-20(t)	Yang et al., 2005
CSL(t + 1) = {1 IF (LIN-12-3(t) AND SEL-8(t) AND LAG-1(t)) OR RAL-1(t) ELSE 0}	Doyle et al., 2000; Greenwald, 2005; Zand et al., 2011
DPY-23(t + 1) = CSL(t)	Yoo et al., 2004
DSL-1(t + 1) = MC(t)	Chen and Greenwald, 2004
EFF-1(t + 1) = {0 IF REF-2(t) OR EGL-18(t) OR ELT-6(t) OR CSL(t) ELSE 1}	Alper and Kenyon, 2002; Shemer and Podbilewicz, 2002; Shemer et al., 2004; Podbilewicz, 2006; Podbilewicz et al., 2006; Alper and Podbilewicz, 2008; Rasmussen et al., 2008
EGL-17(t + 1) = {1 IF LIN-39a(t) == 2, ELSE 0}	Burdine et al., 1997, 1998; Cui and Han, 2003
EGL-18(t + 1) = {1 IF (LIN-39a(t) == 2) ELSE 0}	Koh et al., 2002, 2004; Alper and Podbilewicz, 2008
ELT-6(t + 1) = {1 IF (LIN-39a(t) == 2) ELSE 0}	Koh et al., 2002, 2004; Alper and Podbilewicz, 2008
GAP-1(t + 1) = GAP-1(t)	Hajnal et al., 1997
GSK-3(t + 1) = KIN-19(t) AND APR-1(t) AND PRY-1(t)	Peters et al., 1999; Hoier et al., 2000; Korswagen et al., 2002; Eisenmann, 2005; Oosterveen et al., 2007
HOXCO(t + 1) = {1 IF CEH-20(t) AND UNC-62(t) AND (NOT(CEH-13(t) OR MAB5(t)) OR LIN-39(t) == 2), ELSE 0}	Yang et al., 2005; Tihanyi et al., 2010; Pénigault and Félix, 2011b
KIN-19(t + 1) = APR-1(t)	Peters et al., 1999; Eisenmann, 2005
KSR-1(t + 1) = KSR-1(t)	Roy et al., 2002; Ohmachi et al., 2002
KSR-2(t + 1) = KSR-2(t)	Roy et al., 2002; Ohmachi et al., 2002
LAG-1(t + 1) = LAG-1(t)	Christensen et al., 1996; Doyle et al., 2000
LAG-2(t + 1) = {1 IF MC(t) OR (LIN-39a(t) > 0), ELSE 0}	Chen and Greenwald, 2004; Takács-Vellai et al., 2007; Zhang and Greenwald, 2011
LET-23(t + 1) = {3 IF LET-23ML(t) AND (LIN-3^*^(t) == 3), 2 IF LET-23ML(t) AND LIN-3^*^(t) == 2) AND NOT( ARK-1(t)), 1 IF (ARK-1(t) AND LET-23ML(t) AND LIN-3^*^(t) == 2) OR (LET-23ML(t) AND LIN-3^*^(t) == 1) ELSE 0}	Ferguson and Horvitz, 1985; Aroian and Sternberg, 1991; Kaech et al., 1998; Hopper et al., 2000; Schlessinger, 2000; Worby and Margolis, 2000; Sundaram, 2006
LET-23ML(t + 1) = LIN-2(t) AND LIN-7(t) and LIN-10(t)	Simske et al., 1996; Kaech et al., 1998
LET-60(t + 1) = SOS-1(t)	Han et al., 1990; Hajnal et al., 1997; Chang et al., 2000
LIN-1(t + 1) = LIN-1(t)	Ferguson and Horvitz, 1985; Beitel et al., 1995; Jacobs et al., 1998; Tan et al., 1998; Tiensuu et al., 2005
LIN-10(t + 1) = LIN-10(t)	Ferguson and Horvitz, 1985; Kaech et al., 1998
LIN-11(t + 1) = CSL(t)	Ferguson and Horvitz, 1985; Gupta et al., 2003; Chen and Greenwald, 2004
LIN-12(t + 1) = {1 IF (CSL(t) == 1 OR LIN-39a(t) ≥ 1) ELSE 0}	Greenwald et al., 1983; Christensen et al., 1996; Takács-Vellai et al., 2007
LIN-12-1(t + 1) = {1 IF (LIN-12(t) == 1 AND LIN-14(t) == 0 AND (MC(t) == 0 OR VAV-1(t) == 0)) ELSE 0}	Shaye and Greenwald, 2002, 2005; Yoo and Greenwald, 2005; Li and Greenwald, 2010
LIN-12-2(t + 1) = {1 IF (LIN-12-1(t) AND SUP-17(t) AND LS(t) ) ELSE 0}	Wen et al., 1997; Jarriault and Greenwald, 2005
LIN-12-3(t + 1) = {1 IF (LIN-12-2(t) AND SEL-12(t)) ELSE 0}	Westlund et al., 1999; Goutte et al., 2000; Levitan et al., 2001; Francis et al., 2002; Greenwald, 2005
LIN-14(t + 1) = NOT(lin-4(t))	Li and Greenwald, 2010
LIN-17(t + 1) = CWN-1^*^(t) OR CWN-2^*^(t) OR LIN-44^*^(t)	Ferguson and Horvitz, 1985; Sawa et al., 1996; Deshpande et al., 2005; Gleason et al., 2006; Green et al., 2008
LIN-18(t + 1) = MOM-2^*^(t) OR CWN-2^*^(t)	Ferguson and Horvitz, 1985; Inoue et al., 2004; Deshpande et al., 2005; Green et al., 2008
LIN-2(t + 1) = LIN-2(t)	Ferguson and Horvitz, 1985; Kaech et al., 1998
LIN-25(t + 1) = {1 IF MPK-1(t) == 2, ELSE 0}	Ferguson and Horvitz, 1985; Tuck and Greenwald, 1995; Nilsson et al., 1998
LIN-31(t + 1) = LIN-31(t)	Ferguson and Horvitz, 1985; Tan et al., 1998; Miller et al., 2000; Wagmaister et al., 2006a
LIN-39(t + 1) = {2 IF (PJW5(t) == 2) AND (POP-1b(t) == 1) AND (LIN-39a(t) ≥ 1) AND (MC(t) == 1), 1 IF ((POP-1b(t) == 1 OR PJW5(t) ≥ 1 OR (LIN39a(t) == 2 AND MC(t))) AND NOT ((PJW5(t) == 2) AND (POP-1b(t) == 1) AND (LIN-39a(t) ≥ 1) AND (MC(t) == 1)), ELSE 0}	Bürglin and Ruvkun, 1993; Clark et al., 1993; Tan et al., 1998; Maloof and Kenyon, 1998; Shemer and Podbilewicz, 2002; Wagmaister et al., 2006a,b
LIN-39a(t + 1) = {2 IF MPK-1(t) ≥ 1 AND LIN-39(t) == 2 AND HCO(t), 1 IF NOT (MPK-1(t) ≥ 1 AND LIN-39(t) == 2 AND HCO(t)) AND (LIN39(t) ≥ 1 AND HCO(t)), ELSE 0}	Sternberg and Horvitz, 1986; Eisenmann et al., 1998; Grandien and Sommer, 2001; Yang et al., 2005; Wagmaister et al., 2006b
lin-4(t + 1) = lin-4(t)	Ferguson and Horvitz, 1985; Lee et al., 1993; Li and Greenwald, 2010
LIN-45(t + 1) = {3 IF LET-60(t) == 3, 2 IF LET-60(t) == 2, 1 IF LET-60(t) == 1, ELSE 0}	Han et al., 1993; Hsu et al., 2002
LIN-7(t + 1) = LIN-7(t)	Ferguson and Horvitz, 1985; Kaech et al., 1998
LIP-1(t + 1) = CSL(t)	Berset et al., 2001; Yoo et al., 2004
LIT-1(t + 1) = LIT-1(t)	Kaletta et al., 1997; Siegfried and Kimble, 2002; Lo et al., 2004; Takeshita and Sawa, 2005; Green et al., 2008
LS(t+1) = APX-1^*^(t) OR DSL-1^*^(t) OR LAG-2^*^(t)	Chen and Greenwald, 2004
LST-1(t + 1) = CSL(t)	Yoo et al., 2004
LST-2(t + 1) = CSL(t)	Yoo et al., 2004
LST-3(t + 1) = CSL(t)	Yoo et al., 2004
LST-4(t + 1) = CSL(t)	Yoo et al., 2004
MAB-5(t + 1) = {1 IF EGL-20^*^(t) == 2, ELSE 0}	Bürglin and Ruvkun, 1993; Harris et al., 1996; Maloof and Kenyon, 1998; Alper and Kenyon, 2002; Korswagen et al., 2002; Yuan Jiang and Liu, 2009
MC(t + 1) = LIN-25(t) AND SUR-2(t)	Nilsson et al., 1998
MEK-2(t + 1) = {3 IF (KSR-1(t) OR KSR-2(t)) and (LIN-45(t) == 3), 2 IF (NOT (KSR-1(t) OR KSR-2(t)) AND (LIN-45(t) == 3)) OR (KSR-1(t) OR KSR-2(t)) AND (LIN-45(t) == 2), 1 IF (NOT (KSR-1(t) OR KSR-2(t)) AND (LIN-45(t) == 2)) OR (KSR-1(t) OR KSR-2(t)) AND (LIN-45(t) == 1), ELSE 0}	Wu et al., 1995; Lackner and Kim, 1998; Sundaram, 2006
MIG-1(t + 1) = {1 IF (LIN-44^*^(t) == 1 OR MOM-2^*^(t) == 1) ELSE 0}	Gleason et al., 2006
MIG-5(t + 1) = LIN-17(t) OR MOM-5(t)	Walston et al., 2006
mir61(t + 1) = CSL(t)	Yoo and Greenwald, 2005
MOM-5(t + 1) = CWN-1^*^(t) OR (EGL-20^*^(t) > 0)	Park et al., 2005; Gleason et al., 2006
MPK-1(t + 1) = {2 IF (MEK-2(t) == 3 OR (MEK-2(t) == 2 AND LIP-1(t) == 0)), 1 IF((MEK-2(t) == 2 AND LIP-1(t) == 1) OR (MEK-2 == 1 AND LIP-1(t) == 0)), ELSE 0}	Lackner and Kim, 1998; Berset et al., 2001
PEN-2(t + 1) = PEN-2(t)	Francis et al., 2002
PJW5(t + 1) = {2 IF (LIN-31(t) == 1 AND LIN-1(t) == 1 AND MPK-1(t) ≥ 1), 1 IF LIN-1(t) == 0 AND MPK1(t) ≥ 1, ELSE 0}	Wagmaister et al., 2006a
POP-1(t + 1) = WRM-1(t) AND LIT-1(t)	Lin et al., 1995; Lo et al., 2004
POP-1b(t + 1) = POP-1(t) AND (SYS-1(t) OR BAR-1(t))	Eisenmann, 2005; KiddIII et al., 2005; Phillips et al., 2007; Green et al., 2008
PRY-1(t + 1) = APR-1(t)	Korswagen et al., 2002
RAL-1(t + 1) = RGL-1(t)	Zand et al., 2011
REF-2(t + 1) = {1 IF (LIN-39 ≥ 1), ELSE 0}	Alper and Kenyon, 2002; Alper and Podbilewicz, 2008
RGL-1(t + 1) = {1 IF (LET-60(t) == 1), ELSE 0}	Zand et al., 2011
SEL-12(t + 1) = APH-1(t) AND APH-2(t) AND PEN-2(t)	Westlund et al., 1999; Goutte et al., 2000; Levitan et al., 2001; Francis et al., 2002; Goutte et al., 2002; Greenwald, 2005
SEL-8(t + 1) = SEL-8	Doyle et al., 2000; Petcherski and Kimble, 2000; Greenwald, 2005
SEM-5(t + 1) = LET-23(t)	Clark et al., 1992; Hopper et al., 2000; Worby and Margolis, 2000
SOS-1(t + 1) = SEM-5(t)	Chang et al., 2000; Worby and Margolis, 2000
SUP-17(t + 1) = SUP-17(t)	Jarriault and Greenwald, 2005
SUR-2(t +1) = {1 IF (MPK-1(t) == 2) ELSE 0}	Singh and Han, 1995; Nilsson et al., 1998; Berset et al., 2001
SYS-1(t +1) = NOT (GSK-3(t))	Eisenmann, 2005; KiddIII et al., 2005; Phillips et al., 2007; Green et al., 2008
UNC-62(t + 1) = UNC-62(t)	Yang et al., 2005; Wagmaister et al., 2006a; Pénigault and Félix, 2011b
VANG-1(t + 1) = VANG-1(t)	Green et al., 2008
VAV-1(t + 1) = NOT( mir-61(t))	Yoo and Greenwald, 2005
WRM-1(t + 1) = (NOT GSK-3(t)) AND (LIN-17(t) OR LIN-18(t) OR CAM-1(t)	Lo et al., 2004; Takeshita and Sawa, 2005; Green et al., 2008

**Table A2:** **The simulated effect of different mutations**.

**Case**	**1**	**2**	**VPC**	**F**	**Muv**	**Vul**	**Egl**	**Wild type**	**Mutants**
1	0	0	0	0	0	1	1	0	Mutants in this category imply that the Pn.p cells did not form
2	0	0	0	1	0	1	1	0	Mutations that cause EFF-1 to be constitutively active: *eff1(1), CSL(0);ref-2(0);elt-6(0); egl-18(0)*
3	0	0	1	0	0	1	1	0	
4	0	0	1	1	0	1	1	0	
5	0	1	0	0	1	0	1	0	Ras mutations upstream of RGL-1 which cause RGL-1 to be activated:*let23(1), let-60(1), lin-3^*^*(1), sem-5(1), sos-1(1)
6	0	1	0	1	0	1	1	0	No Ras signaling and no WNT signaling or complete loss of *lin-39* activity: *lin-3(0);cwn-1^*^*(0);cwn-2^*^(0);egl-20^*^(0);lin-44^*^(0);mom-2^*^(0), lin-39(0)
7	0	1	1	0	0	1	1	0	Mutations that do not allow *lin-39* to be completly activated: *ceh-13(1), ceh-20(0), egl-20^*^*(2), let-23(0), let-23ml(0), let-60(0), lin-1(0), lin-10(0), lin-2(0), lin-25(0), lin-3(0), lin-31(0), lin-39(1), lin-39a(0), lin-39a(1), lin-45(0), lin-45(1), lin-7(0), mab-5(1), mc(0), mek-2(0), mek-2(1), mpk-1(0), mpk-1(1), pjw5(0), pjw5(1), sem-5(0), sos-1(0), sur-2(0), unc-62(0)
8	0	1	1	1	0	1	1	0	Loss of Wnt signaling: *apr-1(1), cwn-1^*^*(0);cwn-2^*^(0);egl-20^*^(0);lin-44^*^(0);mom-2^*^(0), gsk-3(1), lit-1(0), mig-5(0), pop-1(0), pop-1b(0), wrm-1(0)
9	1	0	0	0	1	0	1	0	Mutations in Ras signaling genes that cause phosphorilated *lin-39* to remain highly active: *let-23(3), let-60(3), lin-3^*^*(3), lin-39a(2), lin-45(3), mek-2(3), mpk-1(2), sem-5(3), sos-1(3)
10	1	0	0	1	1	1	1	0	
11	1	0	1	0	1	0	1	0	Loss of NOTCH signaling or loss of Ras regulation in second fate cells: *csl(0), lin-11(0), lip-1(0)*
12	1	0	1	1	1	1	1	0	
13	1	1	0	0	1	0	1	0	DSL ligands present in the environment, or mutations that cause Ras signaling to be too active to guarantee the determination of the second fate but not high enough to cause all cells to acquire the first fate: *apx-1^*^*(0);dsl-1^*^(0);lag-2^*^(0);lin-3^*^(2), apx-1^*^(1), dsl-1^*^(1), lag-2^*^(1), csl(1), let-23(2), let-60(2), lin-12_2(1), lin-12_3(1), lin-3(2), lin-45(2), mek-2(2), ral-1(1), rgl-1(1), sem-5(2), sos-1(2)
14	1	1	0	1	0	1	0	0	Loss of *ref-2* fusion control: *ref-2(0)*
15	1	1	1	0	0	0	0	1	*apx-1^*^*(0), cwn-1^*^(0), cwn-2^*^(0), dsl-1^*^(0), lag-2^*^(0), lin-44^*^(0), mom-2^*^(0), aph-1(0), aph-2(0), aph-2(1), apr-1(0), apx-1(0), apx-1(1), ark-1(0), ark-1(1), bar-1(0), bar-1(0);sys-1(1), bar-1(1), cam-1(0), cam-1(1), dpy-23(0), dpy-23(0);gap-1(0), dsl-1(0), dsl-1(1), eff-1(0), egl-17(0), egl-17(1), egl-18(0), egl-18(0);elt-6(0), egl-18(1), egl-20^*^(0), elt-6(0), elt-6(1), gap-1(0), gsk-3(0), kin-19(0), kin-19(1), ksr-1(0), ksr-1(0);ksr-2(0), ksr-2(0), lag-1(0), lag-2(0), lag-2(1), lin-11(1), lin-12(0), lin-12(1), lin-12_1(0), lin-12_1(1), lin-12_2(0), lin12-_3(0), lin-14(0), lin-14(1), lin-17(0), lin-17(0);mig-1(0), lin-17(1), lin-18(0), lin-18(1), lin-25(1), lin-39(2), lin-4(0), lip-1(1), LS(0), lst-1(0), lst-2(0), lst-3(0), lst-4(0), mab-5(0), mc(1), mig-1(0), mig-1(1), mig-5(1), mir-61(0), mir-61(1), mom-5(0), mom-5(1), pen-2(0), pjw5(2), pop-1(1), pop-1b(1), pry-1(0), pry-1(1), ral-1(0), ref-2(1), rgl-1(0), rVPCwt23h, sel-12(0), sel-12(1), sel-8(0), sup-17(0), sur-2(1), sys-1(0), sys-1(1), vang-1(0), vav-1(0), vav-1(1), wrm-1(1)
16	1	1	1	1	1	1	0	0	

*For each mutant we obtained the cellular fates that are represented by the attractors, based on this we classified the mutants, obtaining 16 cases, and for each case we deduced the most likely phenotypes. If a worm does not have first or second fate cells, then it produces a defective vulva an it is very likely to be Egl (Cases 5-12). If the worm has no first and no second fate vulval cells then it will present a Vul phenotype (Cases 1-4). A worm which has no first fate vulval cells is also likely to be Vul (Cases 6, 7, 8), because the determination of the second fate usually depends on first fate cells, unless the second fate is determined by an alternative path. If the fusion fate is active, the worm is Vul (Even cases), a Muv phenotype is expected when only the first or second fates are possible (Cases 5, 9 and 13), when the first fate is possible but the second fate is not (Cases 9-12) Muv and Egl phenotypes are expected*.

### Reasons for the discrepancies between our model and the experimentally reported phenotypes

In *bar-1(0)* mutants, P3.p and P4.p almost always adopt the F fate, while P5.p, P6.p, P7.p, and P8.p adopt the F fate less frequently. Our model is deterministic, and *sys-1* may replace *bar-1* functionally.

The double mutant *elt-6(0);egl-18(0)* is usually lethal, but when its effect is limited to the vulva, it causes a Vul phenotype on some of the worms, in our model *ref-2* and the CSL complex control cell fusion in these mutants, leading to a wild type phenotype.

*ksr-1(0);ksr-2(0)* double mutants and *lip-1(1)* mutants have a Vul phenotype, which our model does not reproduce when a very high level of inductive signal is allowed.

Some *ceh-20(0)* mutant worms have VPCs to be induced (normally, they are not induced), and P5–7.p not to be induced, originating a Muv and Egl phenotype. In our model, *ceh-20(0)* causes a Vul phenotype since first fate cells do not form.

48% of *dpy-23(0);gap-1(0)* double mutants are Muv, but our model produces a wild type phenotype because the precise target for dpy-23 is unknown, and our model is deterministic.

*lin-1(0)* mutants produce a Muv phenotype, but the first fate does not express *egl-17* as much as the wild type P6.p does. According to our model, in such mutants no cell acquires the first fate, but the VPCs may acquire the second or third fate (Case 7 in Table A2); this may cause a Vul or a Muv phenotype. To determine which phenotype may arise, our model would need to cover the events after the first VPC division. Our model indicates, however, that the vulva will not be functional.

*lin-31(constitutive phosphorylation)* causes a Muv phenotype. In our model this mutation is equivalent to allowing pjw5 to have only two levels of activity, namely 1 or 2, which could cause a Vul, or even a wild type phenotype. In order to simulate the Muv phenotype, our model would need to cover the events after the first VPC division.

*lin-12(1);vav-1(0)* increases the penetrance of the *lin-12(1)* Muv phenotype. In our model, both *lin-12_2(1)* and *lin-12_3(1)*, cause a Muv phenotype, and due to the deterministic nature of the model, the effect is not enhanced by *vav-1(0)*.

*pry-1(0)* cause a Muv phenotype in a small percent of the mutants due to defective Wnt signaling regulation, but our model predicts a wild type. In order to simulate this effect, our model would need to be stochastic, and include the events after the first division of the VPCs.

Allowing RAL-1 to activate the CSL complex independently caused the following mutants to have a wild type phenotype in our model: *aph-1(0), aph-2(0), lag-1(0), lin-12(0), lin-14(1), lin-4(0), pen-2(0), sel-12(0), sel-8(0), and sup-17(0)*.

*eff-1(0)* and *egl-17(0)* cause defects during the morphogenesis of the vulva, but our model does not cover these later stages of development.

Only a small percentage of *mig-5(0)* worms present a Muv phenotype or have an ectopic fusion of P5.p or P7.p with hyp7, most likely because *dsh-1* and *dsh-2* may functionally replace *mig-5*. In our model we did not include *dsh-1* and *dsh-2* because their function during the development of the vulva is not known. According to our model losing *mig-5* function will cause a Vul phenotype.

*ral-1(1)* enhances the *lin-12(1)* Muv, but has never been shown to cause a Muv phenotype by itself. In our model *ral-1* activation allows only for the formation of first and second fate cells, causing a Muv phenotype.

**Table A4:** **The simulated effect of removing each interaction**.

**Case**	**1**	**2**	**VPC**	**F**	**Muv**	**Vul**	**Egl**	**Wild type**	**Interactions**
1	0	0	0	0	0	1	1	0	
2	0	0	0	1	0	1	1	0	
3	0	0	1	0	0	1	1	0	
4	0	0	1	1	0	1	1	0	
5	0	1	0	0	1	0	1	0	
6	0	1	0	1	0	1	1	0	
7	0	1	1	0	0	1	1	0	CEH-20 to HOXCO, HOXCO to LIN-39a, LET-23ML to LET-23, LET-23 to SEM-5, LET-60 to LIN-45, LIN-10 to LET-23M, LIN-1 to PJW5, LIN-25 to MC, LIN-2 to LET-23, LIN-39a to LIN-39, LIN-39 to LIN-39a, LIN-3^*^ to LET-23, LIN-45 to MEK-2, LIN-7 to LET-23ML, MC to LIN-39, MEK-2 to MPK-1, MPK-1 to LIN-25, MPK-1 to LIN-39A, MPK-1 to PJW-5, MPK-1 to SUR-2, PJW5 to LIN-39, SEM-5 to SOS-1, SOS-1 to LET-60, SUR-2 to MC, UNC-62 to HOXCO
8	0	1	1	1	0	1	1	0	LIT-1 to POP-1, MIG-5 to APR-1, POP-1b to LIN-39, POP-1 to POP-1b, WRM-1 to POP-1
9	1	0	0	0	1	0	1	0	
10	1	0	0	1	1	1	1	0	
11	1	0	1	0	1	0	1	0	CSL to LIN-11, CSL to LIP-1
12	1	0	1	1	1	1	1	0	
13	1	1	0	0	1	0	1	0	
14	1	1	0	1	0	1	0	0	LIN-39 to REF-2, REF-2 to EFF-1
15	1	1	1	0	0	0	0	1	APX-1^*^ to LS, CWN-1^*^ to LIN-17, CWN-1^*^ to MOM-5, CWN-2^*^ to LIN-17, CWN-2^*^ to LIN-18, DSL-1^*^ to LS, EGL-20^*^ to CAM-1, EGL-20^*^ to MOM-5, LAG-2^*^ to LS, LIN-44^*^ to LIN-17, LIN-44^*^ to MIG-1, MOM-2^*^ to LIN-18, MOM-2^*^ to MIG-1, APH-1 to APH-2, APH-1 to SEL-12, APH-2 to SEL-12, APR-1 to GSK-3, APR-1 to KIN-19, APR-1 to PRY-1, ARK-1 to LET-23, BAR-1 to POP-1B, CAM-1 to WRM-1, CEH-13 to HOXCO, CSL to ARK-1, CSL to DPY-23, CSL to EFF-1, CSL to LAG-1, CSL to LIN-12, CSL to LST-1, CSL to LST-2, CSL to LST-3, CSL to LST-4, CSL to MIR-61, EGL-18 to EFF-1, EGL-20^*^ to MAB-5, ELT-6 to EFF-1, GAP-1 to LET-60, GSK-3 to BAR-1, GSK-3 to SYS-1, GSK-3 to WRM-1, KIN-19 to GSK-3, KSR-1 to MEK-2, KSR-2 to MEK-2, LAG-1 to CSL, LET-60 to RGL-1, LIN-12_1 to LIN-12_2, LIN-12_2 to LIN-12_3, LIN-12_3 to CSL, LIN-12 to LIN-12_1, LIN-14 to LIN-12_1, LIN-17 to MIG-5, LIN-17 to WRM-1, LIN-18 to WRM-1, LIN-31 to PJW-5, LIN-39a to EGL-17, LIN-39a to EGL-18, LIN-39a to ELT-6, LIN-39a to LAG-2, LIN-39a to LIN-12, LIN-39 to HOXCO, LIN-4 to LIN-14, LIP-1 to MPK-1, LS to LIN-12_1, MAB-5 to HOXCO, MC to APX-1, MC to DSL-1, MC to LAG-2, MC to LIN-12_1, MIR-61 to VAV-1, MOM-5 to MIG-5, PEN-2 to SEL-12, PRY-1 to GSK-3, RAL-1 to CSL, RGL-1 to RAL-1, SEL-12 to LIN-12_3, SEL-8 to CSL, SEM-5 to ARK-1, SUP-17 to LIN-12_2, SYS-1 to POP-1b, VANG-1 to CAM-1, VAV-1 to LIN-12_1, vpcwt23h
16	1	1	1	1	1	1	0	0	

*For each interaction removed, we obtained the cellular fates that are represented by the attractors, based on this we classified the interactions using the procedure described in the caption of Table A2*.
